# Exploring the capacity of smoking cessation services in the Philippines

**DOI:** 10.18332/tpc/214732

**Published:** 2026-01-28

**Authors:** Alen Josef A. Santiago, Samantha J. Ackary, Patrik James D. L. Cabrera, Gabriele Dominique P. Domingo

**Affiliations:** 1 School of Government, Ateneo de Manila University, Quezon City, Philippines; 2 Disease Prevention and Control Bureau, Department of Health, Manila City, Philippines

**Keywords:** tobacco control, smoking cessation, addictive behavior, policy, Philippines

## Abstract

**INTRODUCTION:**

In the Philippines, smoking prevalence among adult smokers is slowly decreasing from 23.8% in 2015 to 19.5% in 2021. Despite this decline, evidence shows that while two-thirds of smokers consider quitting, only half receive quit advice from healthcare providers. This study aims to assess the readiness and capacity of health facilities to deliver smoking cessation services in alignment with Administrative Order (AO) 2021-0031 of the Department of Health (DOH).

**METHODS:**

This study followed a cross-sectional design. From August to September 2023, we administered an online questionnaire to health facilities nationwide (n=618) in the Philippines to assess the current status of smoking cessation services across all levels of care regarding physical capacity, technical capacity, and availability of resources.

**RESULTS:**

In this descriptive study of 618 surveyed facilities, only 346 (55.99%) provide smoking cessation services. Among these (n=346), 52.02% (n=180) have certified basic tobacco intervention (BTI) providers, 21.97% (n=75) have certified intensive counseling providers, 88.73% (n=307) screen for tobacco use, 39.60% (n=137) assess for nicotine dependence, 53.18% (n=184) provide intensive counseling, 14.45% (n=50) prescribe pharmacotherapy, 66.47% (n=230) do not have directories or established referral networks, 54.34% (n=188) are aware of the DOH Quitline, and 7.51% (n=26) are aware of mobile-based cessation (mCessation) programs.

**CONCLUSIONS:**

This study highlights the limited capacity of health facilities in the Philippines to deliver smoking cessation services. Our findings suggest several areas for strengthening, including developing cessation infrastructure, expanding designated cessation clinics, targeting awareness campaigns to promote existing services, and expanding access to comprehensive cessation services training programs. Further research can explore and evaluate the effectiveness of these measures to create a stronger basis for resource allocation and policy implementation.

## INTRODUCTION

In the Philippines, tobacco control policies have evolved significantly in alignment with global frameworks such as the World Health Organization (WHO) Framework Convention on Tobacco Control (FCTC)^1^. Significant progress has been made in the past decades, including the passage of smoke-free laws^2^, graphic health warnings on tobacco packaging^3^, tobacco taxation measures^4^, and smoking cessation programs and services^5^. These efforts reflect the country’s commitment to reducing the prevalence of tobacco use and mitigating its associated health impacts in line with international standards. As a result, the Philippines has seen an improvement in terms of diminishing the smoking prevalence among its adults^6,7^. According to the 2021 Global Adult Tobacco Survey^6^, 19.5% (15.1 million) of Filipino adults aged ≥15 years currently use tobacco, a decline from 23.8% of the 2015 GATS^7^. However, the 2021 GATS found that while 63.7% of current smokers planned to or were thinking about quitting, more than half who visited a healthcare provider in the past 12 months received advice to quit smoking.

The WHO FCTC mandates that all its member countries implement measures to promote tobacco cessation and treat nicotine dependence^8^. The Department of Health (DOH), through the Disease Prevention and Control Bureau (DPCB), aims to address the over 20% of non-communicable diseases in the Philippines attributable to tobacco use and reduce its demand as stipulated in the WHO FCTC Article 14. On 5 April 2021, the DOH issued Administrative Order (AO) 2021-0031, entitled ‘Guidelines on the Implementation of Unified and Standardized Tobacco Cessation Services at All Levels of Care’^5^. The issuance outlines the services that should be available at different levels of care and the referral of cases with high nicotine dependence scores. It adopted the recommended actions for developing cessation support, including establishing system components, addressing the issue in healthcare workers, integrating brief advice into existing healthcare systems, and creating capacity for tobacco cessation support and tobacco dependence treatment.

However, the Philippines also faces several challenges in implementing and enforcing smoking cessation policies. These gaps include inadequate funding and resources for tobacco control programs, limited capacity for enforcing smoke-free laws, and persistent interference from the tobacco industry^9^. Consequently, these challenges hinder the full realization of the intended impact of smoking cessation policies and impede progress toward achieving national and global tobacco control goals.

Based on the review of the Philippine National Tobacco Control Strategy (NTCS) 2017–2022 by the DOH, there is a need to assess and improve smoking cessation services, advocating for their incorporation into the outpatient benefit package and exploring unconventional service providers^10^. In addition, the more recent National Tobacco Prevention and Control Strategy (NTPCS) 2030 prioritizes the availability and accessibility of smoking and vaping cessation services at all levels of care^11^. Specifically, Key Strategy 4 of the NTPCS 2030 ‘demands strengthening and widening of the delivery of tobacco cessation services … and at the same time improving the demand for quality and comprehensive cessation services’.

This study aims to present the current landscape of smoking cessation services in the Philippines using the results of a nationwide online survey. In particular, this study will map out the capabilities of the health facilities in the Philippines to deliver population-level smoking cessation services in terms of physical capacity, technical capacity, and availability of resources. In addition, this study aims to build and generate local evidence to help inform the DOH, policymakers, and other relevant stakeholders in the infrastructure planning and policy development on smoking cessation in the Philippines.

## METHODS

### Design

We conducted a cross-sectional nationwide tobacco cessation services survey across all public and private health facilities in the Philippines to measure: 1) physical capacity, 2) technical capacity, and 3) availability of resources to deliver smoking cessation services. The DOH DPCB developed the inventory questionnaire administered online by the School of Government, ADMU, to public and private health facilities across all levels of care in the Philippines. The questionnaire has seven sections, namely: 1) general information on the facility, 2) health human resources for smoking cessation services, 3) basic smoking cessation services, 4) intensive counseling services, 5) pharmacotherapy, 6) referral to higher levels of care, and 7) mobile-based cessation (mCessation) services. The project is conducted under the auspices of the Ateneo Policy Center (APC) under the School of Government, ADMU.

To define the various cessation services that are examined in this study, brief tobacco intervention (BTI) refers to a short, personalized interaction with or counseling of every client, regardless of age, who is a current tobacco user or is a non-tobacco user but is exposed to tobacco smoke^5^. Intensive tobacco cessation counseling refers to an advanced level of intervention utilizing motivational interviewing or behavioral counseling requiring multiple sessions, each lasting 30 minutes or more^5^. This intervention may or may not require pharmacotherapy. Next, the DOH Quitline is a mobile-centered support service under the Lung Center of the Philippines that offers intensive tobacco cessation counseling, requiring multiple sessions to help tobacco users quit. Lastly, as defined by the WHO, mobile-based cessation services, or mCessation, are cessation support provided using one- or two-way messaging through SMS or other channels^12^.

All variables in the questionnaire were categorized as demographic (e.g. region, facility type, facility level), human resource (e.g. number of certified BTI providers, number of certified intensive counseling providers), service provision (e.g. screening for tobacco use, nicotine dependence assessment, availability of pharmacotherapy, referral systems), or awareness/uptake (e.g. awareness of DOH Quitline, awareness of mCessation). Quantitative variables such as the number of intensive counseling providers and number of brief tobacco intervention providers were reported in ranges (e.g. 1–5, 6–10, 11–15, >15). Other categorical response options included ‘routinely’, ‘occasionally’, ‘not at all’, where ‘occasionally’ is defined as implementation that is irregular or non-routine.

### Conduct of the online survey

The tobacco cessation services survey is a self-administered online questionnaire on Airtable, administered from 14 August to 13 September 2023. The research team used Airtable, as the secure platform for data collection. Airtable has been through a Service Organization Controls audit (SOC 2 type 2), compliant with ISO/IEC 27001:2022, and adheres to Europe’s General Data Protection Regulation (GPDR) and the United Kingdom’s GPDR^13^.

The participating health facilities were recruited through a department memorandum containing the link to tobacco cessation services survey. The Office of the Secretary, DOH, issued the memorandum to all Centers for Health Development (CHDs) or the DOH Regional Offices in the Philippines, who then extended the survey to local public and private health facilities from primary to tertiary levels. Facilities were included in the analysis if they completed the survey and reported having a designated cessation clinic or provided smoking cessation services. Facilities that did not complete the survey or indicated that they did not provide cessation services and/or have a designated cessation clinic were excluded from further analysis.

The data responses exhibiting problems such as multiple answers per facility, undeclared health facility codes, and respondents whose roles were unrelated to or lacked knowledge about their facility’s cessation services underwent thorough review and validation. No other personal information was collected from the respondents. Moreover, only relevant information on cessation service delivery of the facilities was collected and retained.

### Data analysis

The Based Checklist for Reporting of Survey Studies (CROSS) checklist is used to report the survey results in this manuscript^14^. We generated frequency and percent distributions to describe the survey data. The variables included demographic profile, facility type, and availability of cessation services and their level. We used Google Sheets for analysis.

## RESULTS

### General information on the facilities

We recruited (n=618) public and private healthcare facilities across all regions of the Philippines (Table 1), including 312 (50.49%) from Luzon, 135 (21.84%) from Visayas, and 171 (27.67%) from Mindanao (Supplementary file Table 1); 244 (39.48%) facilities were designated smoking cessation clinics, while 102 (27.27%) were not designated smoking cessation facilities but provided cessation services (Table 1). This resulted in a total of (n=346) facilities that offer cessation services, which will be the focus of this study. Thus, the succeeding sections will collectively refer to these as ‘facilities with smoking cessation services’.

**Table 1:** Breakdown of facilities, Philippines, August–September 2023

*Facility characteristics*	*Category*	*n (%)*
**Facilities by designation**	Designated smoking cessation clinic	244 (39.48)
	Not designated but providing cessation services	102 (27.27)
	No cessation services offered	272 (72.73)
**Facility type**	Public/government	602 (97.41)
	Private	16 (2.59)
**Level of care**	Primary care	564 (91.26)
	Private care facility	54 (8.74)

### Health human resources for smoking cessation services

Table 2 outlines the available health human resources for delivering brief tobacco intervention (BTI) and intensive counseling, including where these human resources obtain their training to deliver such services.

**Table 2:** Health human resources for smoking cessation services, Philippines, August–September 2023

*Cessation service*	*Indicator*	*Category*	*n (%)*
**Brief tobacco intervention**	Facilities with certified BTI providers	Yes	180 (52.02)
		No	166 (47.98)
	Number of BTI providers per facility with certified providers	1–5	158 (87.78)
		6–10	9 (5.00)
		11–15	2 (1.11)
		>15	11 (6.11)
	BTI training source	Department of Health (DOH)	135 (75.00)
		DOH Academy Platform	34 (18.89)
		Non-Government Organization	5 (2.78)
		Other	6 (3.33)
**Intensive counseling**	Facilities with certified intensive counseling providers	Yes	76 (21.97)
		No	270 (78.03)
	Number of intensive counseling providers per facility with certified providers	1–5	67 (88.16)
		6–10	4 (5.26)
		11–15	3 (3.95)
		>15	2 (2.63)
	Intensive counseling training source	Department of Health (DOH)	58 (76.32)
		DOH Academy Platform	6 (7.89)
		Non-Government Organization	7 (9.21)
		Other	5 (6.58)

For BTI, 180 (52.02%) out of 346 facilities that offer cessation services have certified BTI providers. From the 180 facilities with certified BTI providers, 158 (87.7%) have approximately 1–5 providers in their facility (Supplementary file Table 3), where the most common source of training is from the Department of Health.

On the other hand, even fewer facilities have certified intensive counseling providers, with only 76 (21.97%) facilities meeting this indicator. For the 76 facilities with certified intensive counseling providers, the majority (88.16%) have around 1–5 available providers in their facility (Supplementary file Table 3), with training from the Department of Health (76.32%) being the most common source of training.

### Basic smoking cessation services

We examined the following smoking cessation services: tobacco use screening and nicotine dependence assessment, BTI, intensive counseling, pharmacotherapy, and referral systems.

Out of 346 facilities that offer cessation services, 188 (54.34%) routinely screen for tobacco use (Table 3) primarily through paper-based history and physical examination (PE) forms (83.71%) (Supplementary file Table 4), while 60 (17.34%) routinely conduct nicotine dependence assessments (Table 3) primarily using the Fagerström test for nicotine dependence (70.07%) (Supplementary file Table 5). Common barriers for conducting tobacco use screening include limited human resources (60.40%), resistance from tobacco users (44.51%), and lack of awareness (39.02%) (Supplementary file Table 8). Other barriers for conducting nicotine dependence assessments include insufficient training opportunities for healthcare workers (68.50%), limited human resources (55.20%), and lack of awareness (47.11%).

**Table 3:** Service provision, Philippines, August–September 2023

*Cessation service*	*Indicator*	*Category*	*n (%)*
**Screening and assessment**	Tobacco use screening	Yes, routinely	188 (54.34)
		Yes, occasionally	119 (34.39)
		No	39 (11.27)
	Conduct of nicotine dependence assessment	Yes, routinely	60 (17.34)
		Yes, occasionally	77 (22.25)
		No	209 (60.40)
**Brief tobacco intervention**	Brief tobacco intervention implementation	Yes, routinely being implemented for current tobacco user	100 (28.90)
		Yes, but only occasionally/rarely	157 (45.38)
		No	89 (25.72)
**Intensive counseling**	Intensive counseling implementation	Yes, routinely being provided	45 (13.01)
		Yes, but only occasionally/rarely	139 (40.17)
		No, not being provided	162 (46.82)
**Pharmacotherapy**	Pharmacotherapy prescription	Yes	50 (14.45)
		No	296 (85.55)
**Referral systems**	Existence of established referral system	Yes	116 (33.53)
		No	230 (66.47)

Of 346 facilities, 100 (28.90%) routinely implement BTI for current tobacco users, while 89 (25.72%) do not implement it at all (Table 3). Barriers for implementing BTI include insufficient training opportunities for healthcare workers (63.87%), limited human resources (56.07%), and lack of awareness (41.62%) (Supplementary file Table 8).

Almost half (46.82%) of facilities that offer smoking cessation services do not offer intensive counseling. Commonly cited barriers to implementing intensive counseling include insufficient training opportunities (68.50%), limited human resources (57.23%), and lack of awareness (46.52%).

The majority of facilities (85.55%) do not prescribe pharmacotherapy (Table 3). For facilities that do prescribe pharmacotherapy, the most commonly prescribed type is nicotine replacement therapy (78.00%), followed by varenicline (26.00%) and bupropion (2.00%) (Supplementary file Table 10). Of the 50 facilities that prescribe pharmacotherapy (14.45%), most (66.00%) provide it for free (Supplementary file Table 12); however, the majority do not procure it (56.00%). Budget to support pharmacotherapy procurement is commonly sourced from the facility budget (67.86%) (Supplementary file Table 14).

In terms of referral pathways, the majority of facilities (66.47%) do not have an established referral system (Table 3). Hence, most facilities (75.43%) do not refer current tobacco users for intensive counseling and/or pharmacotherapy (Supplementary file Table 15). Facilities that refer current tobacco users often refer them to other government hospitals (44.71%), followed by within the same facility but to a different department (28.24%) (Supplementary file Table 16). Referrals are primarily monitored by patient feedback upon follow-up (51.76%), a BTI registry/smoking cessation client registry (44.71%), or paper-based referral forms (41.18%).

### Mobile-based cessation (mCessation) and national quitline

We asked participants of their awareness and implementation of other smoking cessation initiatives, specifically mobile-based cessation (mCessation) interventions and the national Department of Health quitline.

Almost all facilities with smoking cessation services (92.49%) are unaware of mCessation (Table 4). Of the 26 (7.51%) facilities that are aware of mCessation, almost half (46.15%) implement an mCessation program.

**Table 4:** mCessation and quitline awareness and implementation, Philippines, August–September 2023

*Service*	*Indicator*	*Category*	*n (%)*
**mCessation**	Awareness of mobile-based cessation (mCessation)	Yes	26 (7.51)
		No	320 (92.49)
	Implementation of mCessation efforts	Yes	12 (46.15)
		No	14 (53.85)
**Quitline**	Awareness of the national Department of Health Quitline	Yes	188 (54.34)
		No	158 (45.66)
	Referral to the national Department of Health Quitline	Yes	72 (38.30)
		No	116 (61.70)

Meanwhile, approximately half of facilities with smoking cessation services (54.34%) are aware of the national Department of Health quitline (61.70%), but the majority of facilities do not refer current tobacco users to the quitline (Table 4).

## DISCUSSION

We assessed the current capacity of health facilities in the Philippines to deliver smoking cessation services. Of the 618 facilities surveyed, just over half reported offering cessation services. Among these, the availability of trained providers, intensive counseling, pharmacotherapy, and referral systems was highly limited. While most facilities routinely screened for tobacco use, only a minority conducted nicotine dependence assessments or provided comprehensive counseling and follow-up. Awareness of national cessation initiatives such as the DOH Quitline and mCessation program was also alarmingly low.

### Technical capacity to deliver smoking cessation interventions

Our survey results revealed a technical capacity deficit in providing tobacco cessation services in the Philippines. The respondents indicated insufficient training opportunities, limited human resources, and overwhelming tasks as barriers to providing BTI services. This may be reflected in the results; while most facilities offered BTI for their clients, only a small minority routinely implement it. In addition, we found a shortage of trained professionals capable of providing intensive counseling, which can hamper the equitable, adequate, and accessible delivery of comprehensive cessation support to smokers seeking professional help.

The WHO FCTC reiterates that offering brief advice from healthcare professionals can lead to a 30% increase in success rates for quitting, while more intensive advice enhances the chances of cessation by 84%^15^. Hence, improving professional performance and technical capacity to deliver smoking cessation services is imperative, and requires formal education and training that strengthens the necessary knowledge and skills that can lead to better patient outcomes in smoking cessation^16,17^. According to the WHO, investing in system-level interventions such as continued education and training programs for healthcare professionals is essential to ensure the effective delivery of smoking cessation interventions^18,19^.

### Physical capacity to deliver smoking cessation services

The 2021 GATS reveal that there is a high interest among the country’s 15.1 million smokers to quit, and yet, only half receive advice from a healthcare professional on how to do this^6^. This underscores the importance of having physical infrastructure in place to deliver cessation services. Despite this, our survey results revealed that the physical capacity to deliver smoking cessation services is lacking. Several factors influence this low capacity, namely: the scarcity of designated smoking cessation clinics, the absence of infrastructure such as screening methodologies and referral systems, and low awareness of the national DOH Quitline and mCessation programs.

More than half of the facilities in our study lacked established referral networks or directories, which indicates weak system linkages for connecting patients to cessation services. Although DOH AO 2021-0031 outlines a referral process, our findings suggest that many facilities still lack the infrastructure and capacity to operationalize and sustain it. To truly institutionalize cessation support, the establishment of clear systems that not only identify smokers but also refer them to the appropriate forms of cessation support is necessary. Studies show that proactive referrals through organized referral pathways can encourage participation in cessation services, underscoring the importance of having established referral systems^20,21^.

Moreover, the survey results revealed a low awareness about the DOH Quitline and mCessation programs. This may reflect limited promotion of these interventions and a lack of integration of cessation support with routine health services. Given the scarcity of designated smoking cessation clinics, a viable complement is expanding the utilization of the DOH Quitline and the adoption of mCessation services to provide accessible support to patients. Interventions such as text-messaging programs, especially those that are tailored and personalized to the patient, have been proven to help people quit smoking in both the short- and long-term, with intensive support needed in tandem for the long-term^22,23^.

### Availability of resources to deliver smoking cessation services

Technical and physical capacities to deliver smoking cessation services are non-negotiable and can only be achieved through sufficient investment in tangible and strategic resources. We found that these resources are still not enough to institutionalize cessation support; specifically, human resources for health, pharmacotherapy, and funding.

On human resources for health, we found that there is not only a lack of training opportunities for healthcare providers to deliver cessation services, but there is also a shortage of available staff. The shortage of certified smoking cessation providers highlights the need to invest in training and workforce development. Evidence from Armenia shows that physicians lack formal cessation training, relying instead on postgraduate programs^24^, a situation that may also apply in the Philippines, where many facilities report limited training opportunities. Exploring forms of support, such as incentives or scholarships for professionals to pursue certification in training for cessation services, such as BTI or intensive counseling, may help boost the workforce capacity in this field.

On pharmacotherapy, previous research studies found that quitting can be more successful when patients use both counseling and medications to enhance the success rate^25,26^. Several studies recognize pharmacotherapy as an essential component of tobacco cessation treatment. However, our study revealed a low turnout of affordable pharmacotherapy prescriptions. The decision to quit smoking must be supported by a healthcare system that provides accessible and affordable smoking cessation services^27^ with access to free or affordable nicotine replacement therapy being a key component of this. Thus, the lack of availability and limited prescription of pharmacotherapy, coupled with challenges in procurement and funding as revealed by the results of our study, underscore the need for greater awareness and support for affordable pharmacotherapy among healthcare providers and policymakers, which can enhance access for tobacco users seeking to quit.

On funding, the issuance of DOH AO 2021-0031 already provides a strong foundation for implementing required services across all levels of care and the referral processes for cessation services, aiming to address the issues, challenges, and opportunities exposed^5^. However, there is no specific provision in the issuance detailing the funding sources required to implement the cessation services defined in the policy^5^. Including smoking cessation treatments in insurance coverage by Filipino individuals through PhilHealth or private insurance companies may address the challenges in affordability and access. A study in the United States found that newly insured patients had a higher success rate in quitting smoking over two years of follow-up than those who stayed uninsured^28^.

One way to promote existing cessation interventions is to launch awareness campaigns; however, this necessitates resources for campaign development and implementation. Persistent smokers, or those who do not quit smoking for 30 consecutive days, who express concerns about their future health, have enhanced motivation for quitting, underscoring the significance of augmenting the beliefs and knowledge of those who smoke for behavioral change towards quitting^29^. While smokers seeking help mostly listen to health professionals’ advice in quitting, others still receive help from mass media campaigns^25^. In a study by Bronsema et al.^30^ among cancer patients seeking smoking cessation support, it was found that awareness campaigns resulted in a major increase in referrals for stop-smoking support. This suggests that allocating funding and support for public education is influential in increasing awareness about cessation services and encouraging their utilization.

### Limitations

The study relied on self-reported data, which may be subject to biases such as information bias or misclassification. Information bias may occur if respondents provide inaccurate information unintentionally due to recall errors or social desirability. Misclassification may occur if respondents interpreted the survey questions differently, leading to possible inconsistencies in reporting insights. Additionally, being cross-sectional, this study cannot make any conclusions about causal relationships between or among the variables examined. Moreover, this study demonstrates high generalizability due to the wide reach of the survey tool through a department memorandum issued by the Department of Health. With more than 600 responses garnered across all three island groups and 18 regions in the Philippines, our findings are likely to represent national-level trends in delivering cessation services.

## CONCLUSIONS

This study describes the status of population-level smoking cessation services in the Philippines and proposes strategies to enhance physical and technical capacities, resource availability, and accessibility of such services. While the DOH AO 2021-0031 and NTPCS 2030 Key Strategy 4 offer a foundation for this, our findings indicate that several gaps must be addressed to fully realize these policies. Effectively addressing challenges and capitalizing on opportunities in smoking cessation services demands a comprehensive strategy encompassing training, infrastructure, human resources, and awareness. Further research is needed to assess the viability of existing tobacco cessation services in the Philippines, to guide the scaling up, reinforcement, tailoring, sustainable financing, and introduction of interventions based on a comprehensive evaluation.

## Supplementary Material



## Data Availability

The data supporting this research are available from the authors on reasonable request.
