# Supplementary material for: Exploring the capacity of smoking cessation services in the Philippines
Source: Tob Prev Cessat. 2026 Jan 28;12:10.18332/tpc/214732. doi: 10.18332/tpc/214732 (PMC12848810; doi:10.18332/tpc/214732)
Supplement: Supplementary file 1 [file TPC-12-05-s1.pdf]

Supplemental Material

Table 1. Facility Types Per Region

This study was conducted from August 2023 to September 2023 via an online questionnaire. It included a total population of (N=618) health facilities across the Philippines. The study examined (n=346) health facilities that are either designated smoking cessation clinics or offer smoking cessation services.

Region	Overall		Private Facility		Public / Government Facility	
	Count	Percentage	Count	Percentage	Count	Percentage
BARMM - Bangsamoro Autonomous Region in Muslim Mindanao	14	2.27%	0	0.00%	14	100.00%
CAR - Cordillera Administrative Region	13	2.10%	0	0.00%	13	100.00%
I - Ilocos Region	79	12.78%	12	15.19%	67	84.81%
II - Cagayan Valley	22	3.56%	1	4.55%	21	95.45%
III - Central Luzon	67	10.84%	0	0.00%	67	100.00%
IV A - CALABARZON	30	4.85%	0	0.00%	30	100.00%
IV B - MIMAROPA	32	5.18%	0	0.00%	32	100.00%
IX - Zamboanga Peninsula	22	3.56%	0	0.00%	22	100.00%
NCR - National Capital Region	28	4.53%	0	0.00%	28	100.00%
V - Bicol Region	41	6.63%	0	0.00%	41	100.00%
VI - Western Visayas	13	2.10%	0	0.00%	13	100.00%
VII - Central Visayas	74	11.97%	3	4.05%	71	95.95%
VIII - Eastern Visayas	48	7.77%	0	0.00%	48	100.00%
X - Northern Mindanao	8	1.29%	0	0.00%	8	100.00%

XI - Davao Region	38	6.15%	0	0.00%	38	100.00%
XII - SOCCSKSARGEN	34	5.50%	0	0.00%	34	100.00%
XIII - Caraga	55	8.90%	0	0.00%	55	100.00%
TOTAL	618	100.00%	16	2.59%	602	97.41%

Table 2. Designated Smoking Cessation Clinics Per Region

This study was conducted from August 2023 to September 2023 via an online questionnaire. It included a total population of (N=618) health facilities across the Philippines. The study examined (n=346) health facilities that are either designated smoking cessation clinics or offer smoking cessation services.

Are you a designated smoking cessation clinic?						
Region	Overall		Yes		No	
	Overall	Percentage	Yes	Percentage	No	Percentage
BARMM - Bangsamoro Autonomous Region in Muslim Mindanao	14	2.27%	0	0.00%	14	100.00%
CAR - Cordillera Administrative Region	13	2.10%	5	38.46%	8	61.54%
I - Ilocos Region	79	12.78%	23	29.11%	56	70.89%
II - Cagayan Valley	22	3.56%	12	54.55%	10	45.45%
III - Central Luzon	67	10.84%	19	28.36%	48	71.64%
IV A - CALABARZON	30	4.85%	6	20.00%	24	80.00%
IV B - MIMAROPA	32	5.18%	10	31.25%	22	68.75%
IX - Zamboanga Peninsula	22	3.56%	13	59.09%	9	40.91%
NCR - National Capital Region	28	4.53%	22	78.57%	6	21.43%
V - Bicol Region	41	6.63%	7	17.07%	34	82.93%
VI - Western Visayas	13	2.10%	7	53.85%	6	46.15%
VII - Central	74	11.97%	14	18.92%	60	81.08%

Visayas						
VIII - Eastern Visayas	48	7.77%	31	64.58%	17	35.42%
X - Northern Mindanao	8	1.29%	4	50.00%	4	50.00%
XI - Davao Region	38	6.15%	25	65.79%	13	34.21%
XII - SOCCSKSARGEN	34	5.50%	29	85.29%	5	14.71%
XIII - Caraga	55	8.90%	17	30.91%	38	69.09%
TOTAL	618	100.00%	244	39.48%	374	60.52%

Table 3. Number Of Brief Tobacco Intervention And Intensive Counseling Providers
This study was conducted from August 2023 to September 2023 via an online questionnaire. It included a total population of (N=618) health facilities across the Philippines. The study examined (n=346) health facilities that are either designated smoking cessation clinics or offer smoking cessation services.

**Service**	**Number of providers**	**Count**	**Percentage**
Brief Tobacco Intervention			
	1-5	158	87.78%
	6-10	9	5.00%
	11-15	2	1.11%
	16-20	8	4.44%
	>20	3	1.67%
	**Total**	**180**	**100.00%**
Intensive Counseling			
	1-5	67	88.16%
	6-10	4	5.26%
	11-15	3	3.95%
	16-20	1	1.32%
	21-25	1	1.32%
	**Total**	**76**	**100.00%**

Table 4. Tobacco Screening Incorporation
This study was conducted from August 2023 to September 2023 via an online questionnaire. It included a total population of (N=618) health facilities across the Philippines. The study examined (n=346) health facilities that are either designated smoking cessation clinics or offer smoking cessation services.

How have you incorporated screening for tobacco use?	Count	Percentage
Inclusion of tobacco use in paper-based History and Patient Encounter Form	257	83.71%
Inclusion of tobacco use in Electronic Medical Records	35	11.40%
Other	15	4.89%
**TOTAL**	307	100.00%

Table 5. Nicotine Dependence Assessment Tools
This study was conducted from August 2023 to September 2023 via an online questionnaire. It included a total population of (N=618) health facilities across the Philippines. The study examined (n=346) health facilities that are either designated smoking cessation clinics or offer smoking cessation services.

Tool	Count	Percentage
Fagerström test for nicotine dependence	96	70.07%
Other	41	29.93%
**TOTAL**	**137**	**100.00%**

Table 6. Number Of Clients Served For Routine Brief Tobacco Intervention
This study was conducted from August 2023 to September 2023 via an online questionnaire. It included a total population of (N=618) health facilities across the Philippines. The study examined (n=346) health facilities that are either designated smoking cessation clinics or offer smoking cessation services.

Frequency	Total Number Of Clients Served In The Past Year For Routine Brief Tobacco Intervention	Count	Percentage
**Annually**			
	**1-25**	25	25.00%
	**26-50**	22	22.00%
	**51-100**	23	23.00%
	**101-250**	9	9.00%
	**251-500**	10	10.00%
	**501-1000**	1	1.00%
	**>1000**	6	6.00%
	***0**	4	4.00%
	**TOTAL**	**100**	**100.00%**
**Monthly**			
	**1-25**	70	
	**26-50**	17	
	**51-100**	7	
	**101-250**	0	
	**251-500**	1	
	**>500**	1	
	***0**	4	
	**TOTAL**	**100**	

Table 7. Number Of Clients Served For Intensive Counseling

This study was conducted from August 2023 to September 2023 via an online questionnaire. It included a total population of (N=618) health facilities across the Philippines. The study examined (n=346) health facilities that are either designated smoking cessation clinics or offer smoking cessation services.

Frequency	Total Number Of Clients Served In The Past Year For Intensive Counseling	Count	Percentage

**Annually**			
	**1-25**	16	35.56%
	**26-50**	6	13.33%
	**51-100**	9	20.00%
	**101-250**	2	4.44%
	**251-500**	5	11.11%
	**501-1000**	0	0.00%
	**>1000**	3	6.67%
	***0**	4	8.89%
	**TOTAL**	**45**	**100.00%**
**Monthly**			
	**1-25**	34	75.56%
	**26-50**	5	11.11%
	**51-100**	0	0.00%
	**101-250**	0	0.00%
	**251-500**	1	2.22%
	**>500**	1	2.22%
	***0**	4	8.89%
	**TOTAL**	**45**	**100.00%**

Table 8. Barriers In Implementing Cessation Services

This study was conducted from August 2023 to September 2023 via an online questionnaire. It included a total population of (N=618) health facilities across the Philippines. The study examined (n=346) health facilities that are either designated smoking cessation clinics or offer smoking cessation services.

Barrier*	Tobacco Use Screening	Nicotine Dependence Assessment	Brief Tobacco Intervention	Intensive Counseling
**Lack of awareness**	135 (39.02%)	163 (47.11%)	144 (41.62%)	162 (46.52%)
**Insufficient training opportunities for healthcare**	220 (63.58%)	237 (68.50%)	221 (63.87%)	237 (68.50%)

**workers**				
**Limited human resources**	209 (60.40%)	191 (55.20%)	194 (56.07%)	198 (57.23%)
**Resistance from tobacco users**	154 (44.51%)	150 (43.35%)	141 (40.57%)	129 (37.28%)
**Cultural or social barriers**	60 (17.34%)	68 (19.65%)	74 (21.39%)	67 (19.36%)
**Others**	14 (4.05%)	10 (2.89%)	12 (3.47%)	8 (2.31%)
**None**	27 (7.80%)	23 (6.65%)	28 (8.09%)	29 (8.38%)

*multiple responses allowed

Table 9. Pharmacotherapy Practice Guidelines
This study was conducted from August 2023 to September 2023 via an online questionnaire. It included a total population of (N=618) health facilities across the Philippines. The study examined (n=346) health facilities that are either designated smoking cessation clinics or offer smoking cessation services.This table looks into the 50/364 facilities that prescribe pharmacotherapy.

What practice guidelines do you use?	Count	Percentage
Philippine Clinical Practice Guidelines for the Diagnosis and Management of Nicotine Dependence	43	86.00%
Other	7	14.00%
**TOTAL**	**50**	**100.00%**

Table 10. Type Of Pharmacotherapy Prescribed
This study was conducted from August 2023 to September 2023 via an online questionnaire. It included a total population of (N=618) health facilities across the Philippines. The study examined (n=346) health facilities that are either designated smoking cessation clinics or offer smoking cessation services.This table looks into the 50/364 facilities that prescribe pharmacotherapy.

What type of pharmacotherapy do you provide/prescribe?	Count	Percentage
Nicotine replacement therapy	39	78.00%

Varenicline	13	26.00%
Bupropion	1	2.00%

Table 11. Prescribed Nicotine Replacement Therapy Forms and Dosages

This study was conducted from August 2023 to September 2023 via an online questionnaire. It included a total population of (N=618) health facilities across the Philippines. The study examined (n=346) health facilities that are either designated smoking cessation clinics or offer smoking cessation services.This table looks into the 50/364 facilities that prescribe pharmacotherapy.

What nicotine replacement therapy forms and dosages do you provide/prescribe?	Count	Percentage
Patches (7, 14, 21mg)	8	20.51%
Patches (10, 15mg)	18	46.15%
Gum	8	20.51%
Others (e.g. Lozenges, Pastille, etc.)	5	12.83%
**TOTAL**	**39**	**100.00%**

Table 12. Provision of Free Pharmacotherapy

This study was conducted from August 2023 to September 2023 via an online questionnaire. It included a total population of (N=618) health facilities across the Philippines. The study examined (n=346) health facilities that are either designated smoking cessation clinics or offer smoking cessation services. This table looks into the 50/364 facilities that prescribe pharmacotherapy.

Do you provide free pharmacotherapy for smoking cessation?	Count	Percentage
Yes, it is free	33	66.00%
No, it is out of pocket	17	34.00%
**TOTAL**	**50**	**100.00%**

Table 13. Procurement of Pharmacotherapy

This study was conducted from August 2023 to September 2023 via an online questionnaire. It included a total population of (N=618) health facilities across the Philippines. The study

examined (n=346) health facilities that are either designated smoking cessation clinics or offer smoking cessation services. This table looks into the 50/364 facilities that prescribe pharmacotherapy.

Do you procure pharmacotherapy for smoking cessation in your facility?	Count	Percentage
Yes	22	44.00%
No	28	56.00%
**TOTAL**	**50**	**100.00%**

Table 14. Alternate Funding Sources for Pharmacotherapy

This study was conducted from August 2023 to September 2023 via an online questionnaire. It included a total population of (N=618) health facilities across the Philippines. The study examined (n=346) health facilities that are either designated smoking cessation clinics or offer smoking cessation services. This table looks into the 50/364 facilities that prescribe pharmacotherapy but do not procure pharmacotherapy.

Source	Count	Percentage
Facility Budget	19	67.86%
Donation	5	17.86%
Grants	1	3.57%
*Donation and Facility Budget	2	7.14%
*Grants and Facility Budget	1	3.57%
**TOTAL**	**28**	**100.00%**

Table 15. Referral For Intensive Counseling/Pharmacotherapy

This study was conducted from August 2023 to September 2023 via an online questionnaire. It included a total population of (N=618) health facilities across the Philippines. The study examined (n=346) health facilities that are either designated smoking cessation clinics or offer smoking cessation services.

Do you refer current tobacco users for intensive counseling and/or pharmacotherapy?	Count	Percentage

Yes	85	24.57%
No	261	75.43%
**TOTAL**	**346**	**100.00%**

Table 16. Referral Unit

This study was conducted from August 2023 to September 2023 via an online questionnaire. It included a total population of (N=618) health facilities across the Philippines. The study examined (n=346) health facilities that are either designated smoking cessation clinics or offer smoking cessation services. This table looks into the 85/364 facilities that refer current tobacco users for intensive counseling and/or pharmacotherapy.

**Where do you mainly refer your clients?**	**Count**	**Percentage**
Same facility, different department/office/clinic	24	28.24%
Different facility (Government hospital)	38	44.71%
Different facility (Private hospital/clinics)	1	1.18%
Different facility (Designated smoking cessation clinic)	6	7.06%
National DOH Quitline	16	18.82%
**TOTAL**	**85**	**100.00%**

Table 17. Referral Monitoring

This study was conducted from August 2023 to September 2023 via an online questionnaire. It included a total population of (N=618) health facilities across the Philippines. The study examined (n=346) health facilities that are either designated smoking cessation clinics or offer smoking cessation services. This table looks into the 85/364 facilities that refer current tobacco users for intensive counseling and/or pharmacotherapy.

**How do you monitor the referrals?***	**Count**	**Percentage**
Brief tobacco intervention registry/smoking cessation client registry	38	44.71%
Digital shared information system	6	7.06%
Paper-based referral form with return slip	35	41.18%

Patient feedback upon follow-up	44	51.76%
Others	2	2.35%

*check all that apply

Table 18. Receiving Referrals

This study was conducted from August 2023 to September 2023 via an online questionnaire. It included a total population of (N=618) health facilities across the Philippines. The study examined (n=346) health facilities that are either designated smoking cessation clinics or offer smoking cessation services.

Do you receive referrals from other facilities/places?	Count	Percentage
Yes<br>(Health Facilities, Workplaces, Tuberculosis (TB) Directly Observed Treatment (DOTS) Clinics, Schools, and Department of Health Quitline)	119	34.39%
No	227	65.61%
**TOTAL**	346	**100.00%**

© 2026 Santiago A.J.A. et al.
